# The *Legionella pneumophila* effector PieF modulates mRNA stability through association with eukaryotic CCR4−NOT

**DOI:** 10.1128/msphere.00891-24

**Published:** 2024-12-19

**Authors:** Harley O'Connor Mount, Malene L. Urbanus, Francesco Zangari, Anne-Claude Gingras, Alexander W. Ensminger

**Affiliations:** 1 Department of Molecular Genetics, University of Toronto 7938 , Toronto, Ontario, Canada; 2 Department of Biochemistry, University of Toronto 7938 , Toronto, Ontario, Canada; 3 Lunenfeld-Tanenbaum Research Institute, Sinai Health 518775 , Toronto, Ontario, Canada; University of Kentucky College of Medicine, Lexington, Kentucky, USA

**Keywords:** *Legionella pneumophila*, host-pathogen interaction, deadenylation, CCR4-NOT, mRNA stability, PieF, effectors, CNOT7 inhibition, CNOT7/8, Caf1, Pop2, DEDD

## Abstract

The eukaryotic CCR4−NOT deadenylase complex is a highly conserved regulator of mRNA metabolism that influences the expression of the complete transcriptome, representing a prime target for a generalist bacterial pathogen. We show that a translocated bacterial effector protein, PieF (Lpg1972) of *Legionella pneumophila*, directly interacts with the CNOT7/8 nuclease module of CCR4−NOT, with a dissociation constant in the low nanomolar range. PieF is a robust *in vitro* inhibitor of the DEDD-type nuclease, CNOT7, acting in a stoichiometric, dose-dependent manner. Heterologous expression of PieF phenocopies knockout of the CNOT7 ortholog (POP2) in *Saccharomyces cerevisiae*, resulting in 6-azauracil sensitivity. In mammalian cells, expression of PieF leads to a variety of quantifiable phenotypes: PieF silences gene expression and reduces mRNA steady-state levels when artificially tethered to a reporter transcript, and its overexpression results in the nuclear exclusion of CNOT7. PieF expression also disrupts the association between CNOT6/6L EEP-type nucleases and CNOT7. Adding to the complexities of PieF activity *in vivo*, we identified a separate domain of PieF responsible for binding to eukaryotic kinases. Unlike what we observe for CNOT6/6L, we show that these interactions can occur concomitantly with PieF’s binding to CNOT7. Collectively, this work reveals a new, highly conserved target of *L. pneumophila* effectors and suggests a mechanism by which the pathogen may be modulating host mRNA stability and expression during infection.

**IMPORTANCE:**

The intracellular bacterial pathogen *Legionella pneumophila* targets conserved eukaryotic pathways to establish a replicative niche inside host cells. With a host range that spans billions of years of evolution (from protists to humans), the interaction between *L. pneumophila* and its hosts frequently involves conserved eukaryotic pathways (protein translation, ubiquitination, membrane trafficking, autophagy, and the cytoskeleton). Here, we present the identification of a new, highly conserved host target of *L. pneumophila* effectors: the CCR4−NOT complex. CCR4−NOT modulates mRNA stability in eukaryotes from yeast to humans, making it an attractive target for a generalist pathogen, such as *L. pneumophila*. We show that the uncharacterized *L. pneumophila* effector PieF specifically targets one component of this complex, the deadenylase subunit CNOT7/8. We show that the interaction between PieF and CNOT7 is direct, occurs with high affinity, and reshapes the catalytic activity, localization, and composition of the complex across evolutionarily diverse eukaryotic cells.

## INTRODUCTION

*Legionella pneumophila* is a Gram-negative bacterial species found frequently in freshwater environments (1, 2). Within these environments, *L. pneumophila* replicates intracellularly within diverse protozoan hosts separated by billions of years of eukaryotic evolution (3–5). Humans are typically exposed to *L. pneumophila* through inhalation of microbe-laden water droplets (6, 7) from contaminated water infrastructure, resulting in Legionnaires’ disease (8–14). When *L. pneumophila* encounters human lung alveolar macrophages, a pathogenic program is initiated that is highly similar to that used within protozoan hosts (15–18). In both cases, the bacterium relies on the injection of pathogenic proteins, called “effectors,” through a type IV secretion system (19–21). Collectively, over 300 effectors prevent phagolysosome fusion with the *Legionella*-containing vacuole (LCV) and recruit host factors and membrane constituents to help nourish and accommodate the growing intracellular bacterial population (22). The vast majority of effectors are individually dispensable for intracellular replication, due largely to overlapping host targets and other sources of genetic redundancy (23–26).

Most *L. pneumophila* effectors are not encoded within canonical genomic islands, but rather are found throughout the genome (27). Some, however, are encoded in regions of genomic plasticity that demonstrate variation between *L. pneumophila* strains (28–30). One such plasticity region was previously shown to encode several effectors that vary between *L. pneumophila* isolates and was subsequently named the **p**lasticity **i**sland of **e**ffectors (or *pie* locus) (30). The effectors within the *pie* locus are named *pieA-G* and are interspersed with several non-translocated protein coding genes as well (30). Since the discovery of this locus, PieA, PieE, and PieG have been characterized mechanistically (30–32). PieG (LegG1) is the best characterized and is prenylated at its C-terminus, enabling localization to the LCV where it facilitates LCV motility through activation of host Ran GTPase (32–35). PieE was shown to associate with mammalian Rab GTPases (Rab5 and Rab7) and likely influences vesicular trafficking and GTPase recruitment to the LCV (31). The function of PieA is less obvious, but it localizes to the LCV as well (30). The activities of PieB,-C,-D and -F remain undefined.

Rewiring and silencing host gene expression are often critical mechanisms for the survival and replication of intracellular bacterial pathogens (36). Once inside a host cell, these invaders seek to dampen immune and/or stress response signaling by blocking transcription and translation of host defense factors (*e.g.*, proinflammatory cytokines, pro-apoptotic caspases, positive and negative regulators of immune signaling pathways) (36–41). In addition to blocking host responses, limiting host gene expression can also free up micronutrients like amino acids that may be redirected for bacterial use (42–44). The mechanisms that pathogens use to modulate host gene expression are diverse and largely pathogen-dependent (45). *L. pneumophila* utilizes at least nine effectors to interfere with host translation through diverse mechanisms to alter the cell cycle and influence immune signaling cascades (43, 46–56). Given the large number of *L. pneumophila* effectors that remain to be functionally characterized, it is likely that other pathogenic mechanisms of modulating host gene regulation are yet to be described.

The eukaryotic CCR4−NOT complex is a large (>1 MDa) evolutionarily conserved molecular machine with critical roles in mRNA metabolism (57). The best characterized role of CCR4−NOT is to shorten poly(A) tails of mature mRNA, as one of two major deadenylase complexes of eukaryotic cells (57). The other major deadenylase complex, PAN2/3, initially shortens mRNA poly(A) tails, priming them for the CCR4−NOT complex, which further deadenylates the transcript, promoting either translation repression or decay pathways (for reviews see Ref. [57–60]). CCR4−NOT influences eukaryotic gene expression in concert with other deadenylases and CCR4−NOT-associated factors, allowing for temporal control of mRNA stability and turnover of gene expression programs (57). As a central regulator of eukaryotic gene expression, the CCR4−NOT complex has a critical role in ensuring proper cellular function (57). Given its vital cellular role, it is unsurprising that some subunits of CCR4−NOT are essential for mammalian development and cell viability (61).

The CCR4−NOT complex is scaffolded by the large multi-domain protein CNOT1 (62, 63). The middle domain of eukaryotic initiation factor 4G (MIF4G) is found at the center of CNOT1 and is required for binding the DEDD (Asp-Glu-Asp-Asp) nuclease module (the paralogs CNOT7 and CNOT8 in mammalian cells) (62, 63). CNOT7 binds the MIF4G domain in a mutually exclusive manner with CNOT8, and each subunit carries out similar, but distinct catalytic roles (64). In addition to the DEDD nuclease, CCR4−NOT contains a secondary endonuclease−exonuclease−phosphatase (EEP) nuclease module composed of the paralogs CNOT6 and CNOT6L (65, 66), which directly depend upon the DEDD module for incorporation into the CCR4−NOT complex (67, 68). Despite the catalytic similarity, each nuclease module regulates distinct mRNA subsets (69), with specific poly(A)-binding protein (PABP) occupancy preference (70). CCR4−NOT activity can be directed through the activity of adapter proteins, including the antiproliferative BTG/TOB factors that associate with RNA-binding proteins (*e.g.*, PABPC) to drive CCR4−NOT recruitment to mRNA (71–75).

Here, we show that the *L. pneumophila* effector PieF (Lpg1972) directly binds CNOT7/8 and inhibits its nuclease activity *in vitro*. We show that heterologous expression of PieF leads to a variety of quantifiable phenotypes in both yeast and mammalian cells, including modulation of mRNA stability, CNOT7 localization, and CCR4−NOT complex composition. Collectively, these observations reveal CCR4−NOT modulation as another facet of highly conserved eukaryotic biology that is co-opted during *L. pneumophila* pathogenesis.

## RESULTS

### PieF interacts with the CNOT7/8 subunits of the CCR4−NOT complex

PieF was shown to be a Dot/Icm translocated substrate (30), but its function remains uncharacterized. Like most *L. pneumophila* effectors, loss of PieF does not cause a *Legionella* growth defect as shown by growth of PieF transposon insertion mutants in U937 cells and four protozoan hosts (26). In addition, it has no primary sequence similarity or structural similarity in AlphaFold clusters to proteins with known functions (76) (https://cluster.foldseek.com/cluster/A0A378IXZ9).

**Fig 1:**
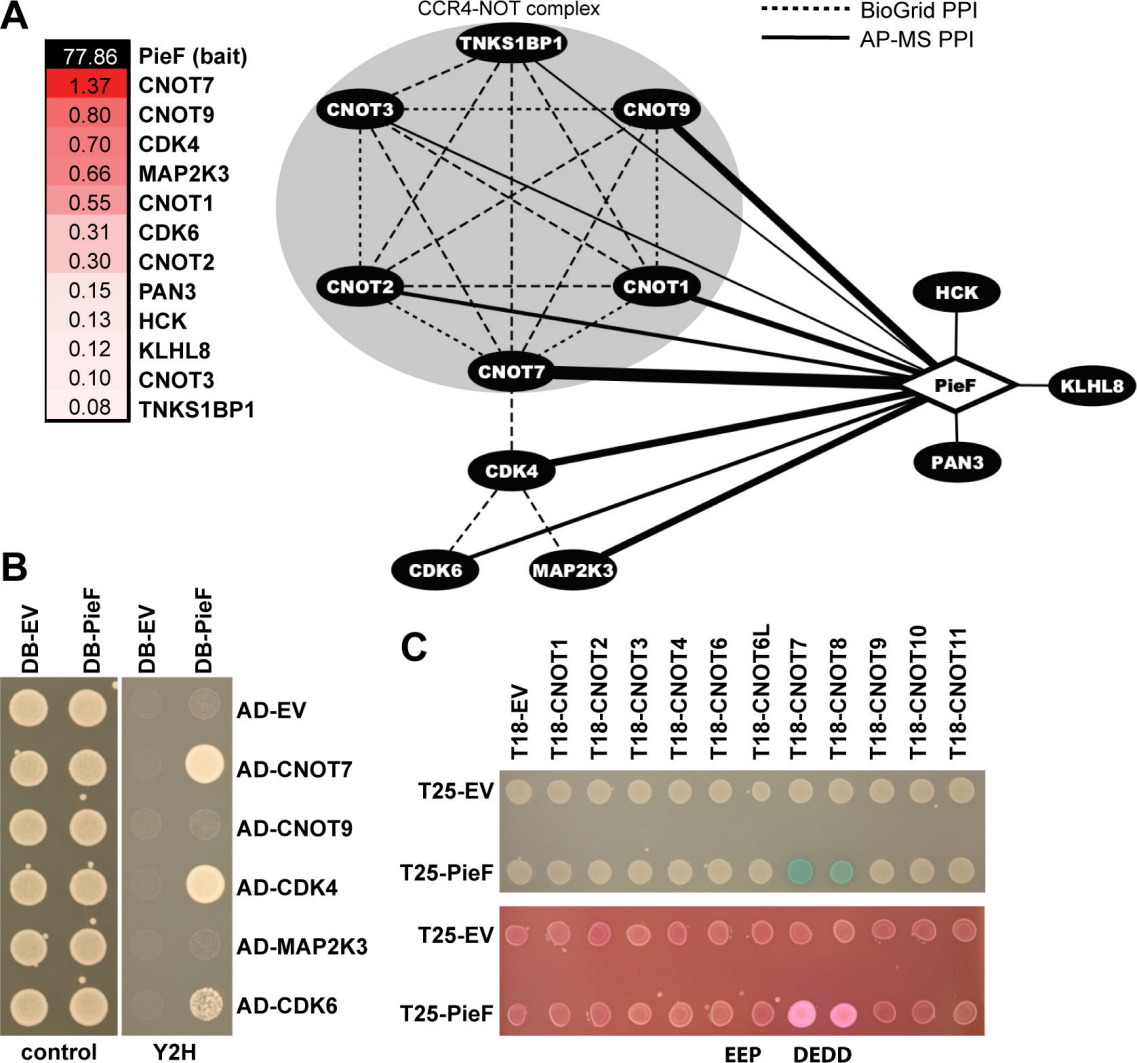
Identifying PieF host targets by AP-MS. (**A**) Purified 6xHis-SBP-PieF was immobilized on streptavidin beads and incubated with U937 lysate. Host proteins co-purifying with PieF were identified using mass spectrometry and are represented in a heatmap showing the normalized spectral counts [peptide count/protein size (kDa)] of the bait (PieF) and prey (host) proteins. Prey proteins with fewer than eight average unique peptides supporting the interaction were filtered. A network representation of the prey proteins shows an enrichment for CCR4−NOT complex components. Network edges represent peptide spectral counts normalized to protein size [spectral counts/size (kDa)]. BioGrid database (https://thebiogrid.org/) interactions are illustrated with dashed edges. (**B**) Confirmation of the top prey proteins by Y2H assay. Yeast strains expressing the GAL4 DNA-binding domain (DB) PieF fusion or empty vector (EV) control and GAL4-activating domain (AD) prey fusions or EV control were stamped onto SC-Leu/Trp (control) or SC-Leu/Trp/His (Y2H) agar plates and grown for 2 days at 30°C. Gal4p transcription factor complementation stimulates expression of reporter gene HIS3 allowing for growth on Y2H selective medium. The experiment was performed in biological triplicate, and a representative example is shown. (**C**) BACTH assay demonstrating a direct physical interaction between PieF and both DEDD nuclease subunits CNOT7 and CNOT8 of the CCR4−NOT complex. Adenylate cyclase complementation stimulates expression of the maltose operon, as well as LacZ induction. Complementation is assayed through cleavage of 5-bromo-4-chloro-3-indolyl-β-D-galactopyranoside (X-gal) by LacZ (upper panel, blue coloring) or growth on MacConkey medium with maltose as the sole carbon source (lower panel, pink coloring). The experiment was performed in biological triplicate, and a representative example is shown.

To identify the host target(s) of PieF, we used affinity purification coupled with mass spectrometry (AP-MS) as described previously (77, 78). Purified 6xHis-SBP-tagged PieF was immobilized on streptavidin beads, incubated with human U937 lysate and copurifying host proteins were identified using mass spectrometry (see Materials and Methods). Of the 12 proteins that copurify with PieF, five are components of the CCR4−NOT complex (CNOT1, CNOT2, CNOT3, CNOT7, and CNOT9) (79–81), while the 182 kDa tankyrase-1-binding protein (TNKS1BP1) is an CCR4−NOT-associated factor (64). Two cyclin-dependent kinases, CDK4 and CDK6 also co-purified with PieF. When normalized for protein size, however, the top hit was CNOT7–the DEDD-type deadenylase subunit of the CCR4−NOT complex (Fig. 1A).

The enrichment of several CCR4−NOT components suggested that one or more of these interactions with PieF was likely indirect. To test this, we performed the yeast-two hybrid (Y2H) assay between each of the top AP-MS hits and PieF, which confirmed interactions between PieF and CNOT7, CDK4, and CDK6 (Fig. 1B).

While Y2H generally enriches for direct interactions, because the CCR4−NOT complex is highly conserved (with endogenous orthologs *in S. cerevisiae*), we next turned to an assay where direct interactions could be assessed without this potentially complicating factor. An orthologous protein−protein interaction method, the bacterial adenylate cyclase two-hybrid system (BACTH) is performed in *Escherichia coli* (82, 83)*,* which does not have a CCR4−NOT complex. As such, a positive interaction signal using this assay would indicate a direct and binary interaction assay between PieF and individual CCR4−NOT components. This assay confirmed an interaction between PieF and CNOT7, as well as a close paralog of CNOT7 (CNOT8) (Fig. 1C). Taken together, these results are consistent with CNOT7/8, the DEDD-type deadenylase subunit of CCR4−NOT, serving as the direct target of PieF, with the other CCR4−NOT components co-purifying with PieF due to their association with CNOT7 (see Fig. 1A, BioGRID database interactions [84]).

### A comprehensive yeast two-hybrid screen between effectors and several CCR4−NOT components suggests that the PieF−CNOT7/8 interaction is unique

**Fig 2:**
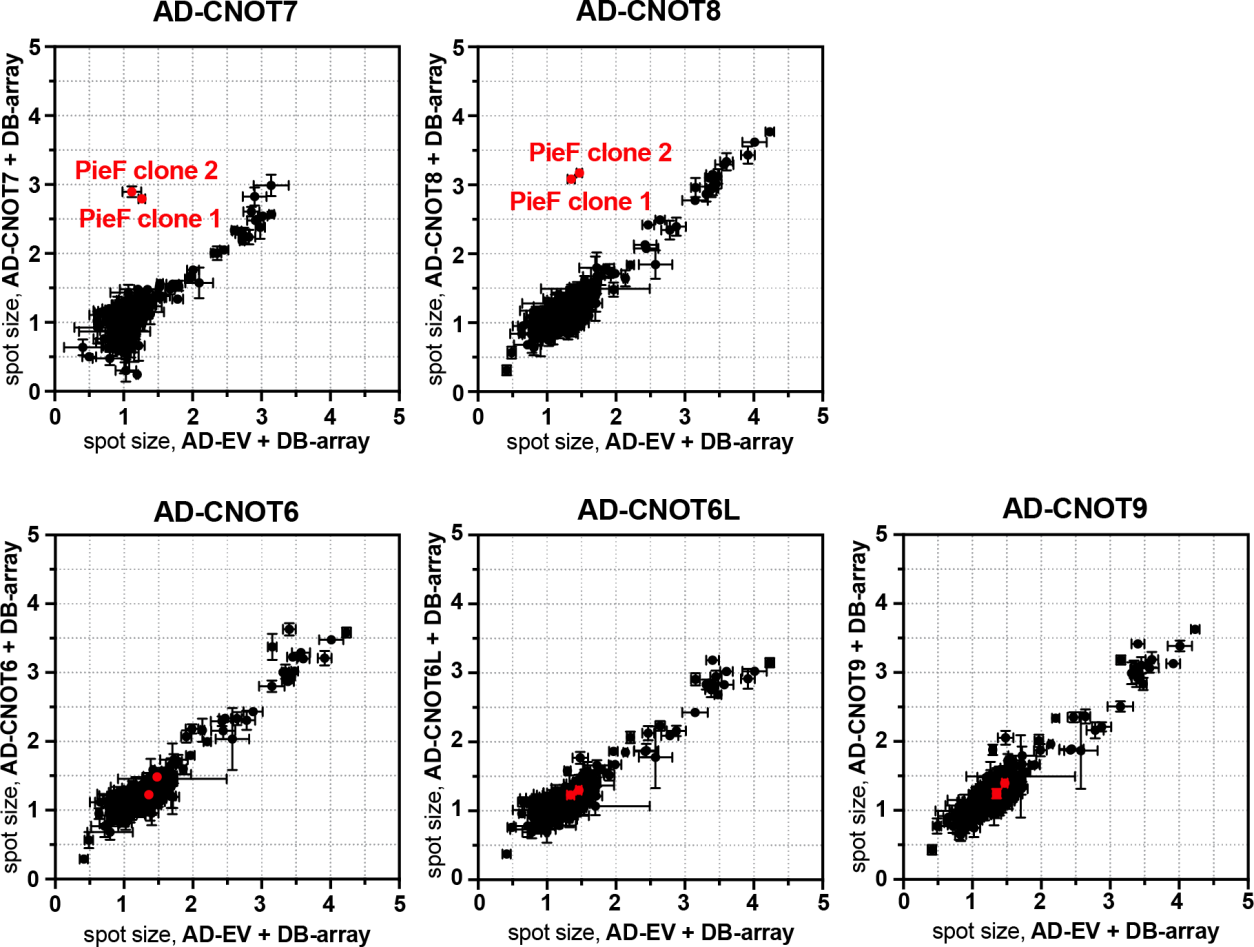
PieF is the only effector with a positive Y2H signal with the CCR4−CNOT complex. A Y2H screen of AD-tagged CNOT6, 6L, 7, 8, and 9 against DB-tagged *L. pneumophila* str. Philadelphia effectors. Haploid yeast strains carrying a library of 348 DB-tagged effectors and putative effectors in a copper-inducible vector were arrayed in 1,536-format, where each effector was represented by two clones, and each clone is present in quadruplicate. The arrayed strains were mated with a compatible haploid yeast strain carrying AD-tagged CCR4−CNOT complex component or an empty vector control (EV). The diploid arrays were assayed for growth on Y2H selective medium (lacking tryptophan, leucine, and histidine) with copper to induce expression of the DB-effector fusions. Spot sizes of strains co-expressing the AD-tagged bait clone and each of the 348 effectors within the arrayed DB-effector library were quantified, normalized against DB-empty vector controls on the array and plotted as AD-CNOT + DB-array diploids (y-axis) vs AD-empty vector + DB-array diploids (x-axis). Error bars represent standard deviation (SD) of spot size between quadruplicate spots within each data set. A positive Y2H signal is revealed as a deviation above the diagonal, and PieF is shown in red.

Given that *L. pneumophila* often targets host processes with multiple effectors (*e.g.*, at least nine effectors influence protein translation during infection [38, 39, 43, 47, 53, 54, 56]), we next determined whether multiple effectors might target CNOT7/8, or other subunits of CCR4−NOT. We performed a comprehensive yeast two-hybrid analysis using the two DEDD-type deadenylases (CNOT7 and CNOT8), the EEP-type deadenylases (CNOT6 and CNOT6L), and another CCR4−NOT subunit (CNOT9) as bait. Notably, among the 348 effectors (and putative effectors) in the library, PieF was the only protein observed to interact with the DEDD-type deadenylases CNOT7 and CNOT8 (Fig. 2, top row). In contrast, no effectors (including PieF) were observed to interact with the EEP-type deadenylase CNOT6, CNOT6L, or CNOT9 (Fig. 2, bottom row). Notably, the specificity of the PieF−CNOT7 interaction is consistent with the results from a global effector-wide interaction screen, in which we recently identified several physical interactions between *L. pneumophila* effectors and a positive reference set of human proteins, which fortuitously included CNOT7 (85). One of the most connected nodes within the interaction network was PieF, whose verified interaction partners included CNOT7, LSM3, IKZF1, and CDK4 along with the bacterial effector kinase LegK2 (85). In that screen, like here, no other effectors displayed a positive interaction signal with CNOT7.

### PieF binds CNOT7 at low nanomolar affinity and inhibits CNOT7 deadenylase activity *in vitro*

**Fig 3:**
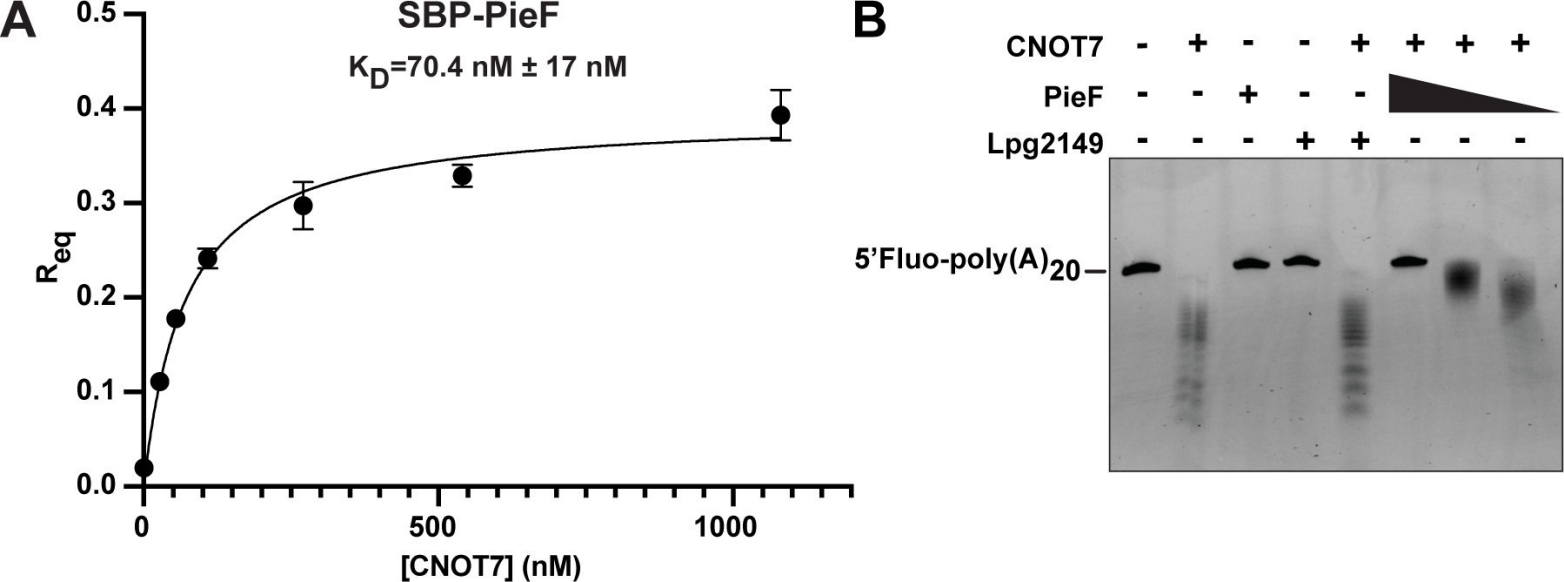
PieF inhibits CNOT7 catalytic activity. (**A**) PieF binds CNOT7 with nanomolar affinity. Binding affinity was measured using biolayer interferometry saturation curves acquired with a Forte-Bio instrument. Binding affinity measurements for CNOT7 against sensor-bound 6His-SBP-PieF were fit using a single site-specific binding model in Prism (Prism 9). Results are representative of three independent experiments. Error bars represent the SEM of three independent experiments. (**B**) PieF impairs the degradation of a synthetic RNA substrate at equimolar ratios with CNOT7, while an unrelated *Legionella* effector Lpg2149 does not. The RNA substrate labeled at the 5' end with 6-carboxyfluorescein and contains a 20 nucleotide poly(A) tail is incubated with purified 1 µM CNOT7, PieF, Lpg2149, or CNOT7 with Lpg2149 or PieF (1, 0.5, or 0.25 µM) before resolving the RNA degradation products on 25% 8M UREA-PAGE.

To measure the binding affinity between PieF and CNOT7, we next performed biolayer interferometry (BLI) kinetic analysis using purified CNOT7 and PieF protein. From this, we calculated the dissociation constant of untagged CNOT7 from PieF as 70.4 nM (±17 nM) (Fig. 3A). To rule out non-specific binding, we also performed a negative control experiment in which untagged CNOT7 was incubated with a bare streptavidin biosensor at the highest tested concentration (1,080 nM). CNOT7 failed to bind the streptavidin biosensor in the absence of PieF (Table S1). Taken together, these results demonstrate that PieF binds CNOT7 protein with a biologically relevant affinity (86). For comparison, PieF may bind CNOT7 more tightly than the BTG/TOB proteins, a family of well-characterized, endogenous CNOT7 interaction partners (87). The dissociation constant between PieF and CNOT7 that we measure is ~5–9 times lower than what has been reported between CNOT7 and TOB by isothermal titration calorimetry (88, 89).

As CCR4−NOT regulates diverse aspects of eukaryotic biology including apoptosis, immune-signaling, and cellular proliferation, identifying a specific output of PieF-mediated modulation represents a significant challenge. After demonstrating a direct physical interaction between PieF and CNOT7, we next investigated the functional output of the PieF−CNOT7 interaction using a well-characterized *in vitro* deadenylase assay (88–93). The best-characterized molecular activity of CNOT7/8 is to deadenylate mRNA transcripts as a 3′−5′ exonuclease (94, 95). This activity can be quantified *in vitro* by incubating purified nucleases activity with 5′-fluorescently labeled, synthetic RNA substrates whose subsequently reduced length can be visualized on a polyacrylamide gel. Using bacterially expressed and purified CNOT7 and PieF, we performed the CNOT7 deadenylase assay in the presence and absence of PieF. Comparable to previous studies, purified CNOT7 showed robust deadenylase activity against a 5′ fluorescently labeled (6-FAM) RNA substrate (88, 91–93), resulting in a characteristic laddering pattern of shortened RNA species (Fig. 3B). On their own, purified PieF and a negative, unrelated effector control, Lpg2149, showed no deadenylase activity against this substrate. (Lpg2149 was chosen as an effector control because it does not target host proteins during infection [96], but its molecular weight closely approximates that of PieF.) When PieF was preincubated with CNOT7 at equimolar ratios, nuclease activity was efficiently blocked (Fig. 3B). No inhibition of CNOT7 was observed after preincubation with the unrelated effector control, Lpg2149. The inhibition of CNOT7 by PieF appears to be stoichiometric, diminishing at molar ratios below 1:1 (Fig. 3B). One explanation is that PieF may sterically occlude the active site of CNOT7 *in vitro*. Alternatively, PieF binding may promote conformational changes in CNOT7, triggering the release of the RNA substrate for another deadenylase (CNOT6/6L), or process such as de-capping (97).

### Heterologous expression of PieF in yeast phenocopies deletion of the CNOT7/8 ortholog, *POP2*

**Fig 4:**
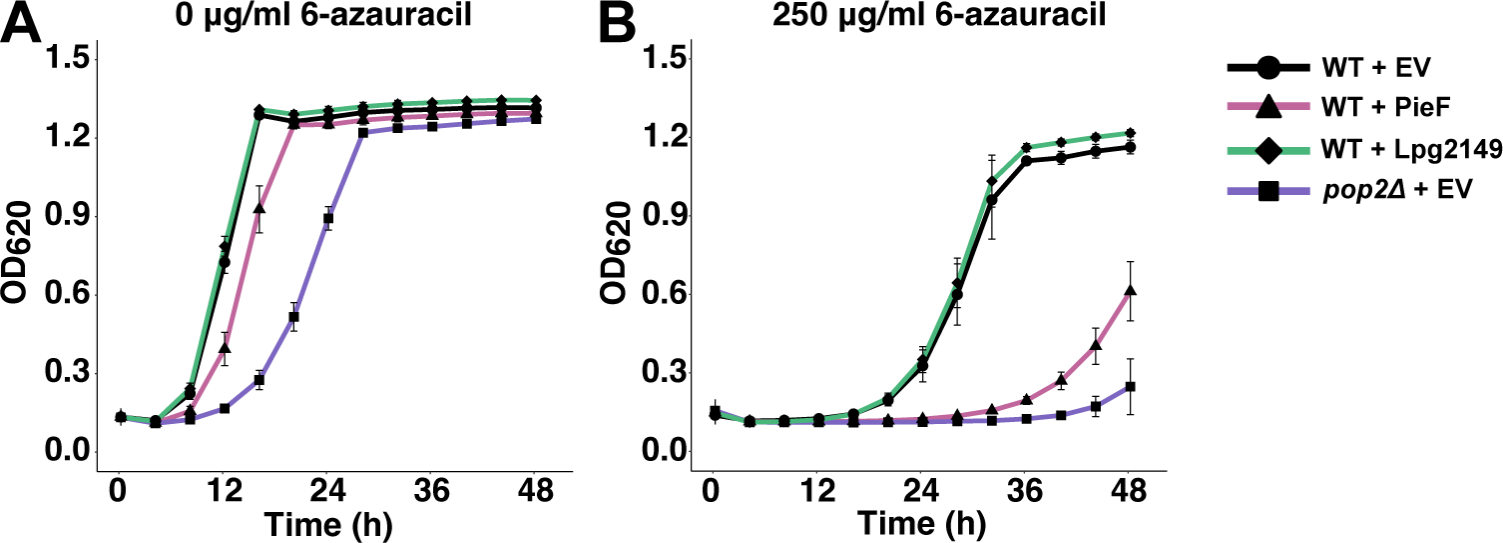
PieF expression phenocopies deletion of *CNOT7* (*POP2*) in yeast. Growth curves of wild-type BY4741 carrying overexpression vectors of PieF, Lpg2149, or empty vector control (EV), and BY4741 *pop2Δ::KANMX* carrying EV control in the absence (**A**) or presence (**B**) of 250 µg/mL 6-azauracil. The average of two experiments and the standard error of the mean are plotted. PieF expression demonstrates a modest growth defect in the absence of 6-azauracil. Deletion of the yeast *CNOT7* ortholog *POP2* leads to an increased growth defect in the presence of 6-azauracil as does expression of PieF, but not with the expression of Lpg2149 or an EV control.

CCR4−NOT was first identified and studied in *S. cerevisiae* (98) and deletion of the yeast ortholog of *CNOT7/8*, *POP2,* leads to several phenotypes, including reduced growth rate in rich medium, and sensitivity to the transcriptional elongation inhibitor 6-azauracil (99). CCR4−NOT components have synthetic lethal genetic interactions with transcriptional elongation factors and can stimulate the elongation of arrested RNA polymerase II *in vitro*, supporting a role in transcriptional elongation (99–102). 6-Azauracil depletes cellular pools of GTP by inhibiting IMP-dehydrogenase, which also blocks transcriptional elongation by RNA polymerase II (103). These established phenotypes provided us with a simple assay for the effects of PieF on CNOT7 (Pop2p) activity inside the eukaryotic cell. We assayed the growth of *S. cerevisiae* strains expressing PieF or lacking *POP2* in the presence of 6-azauracil (Fig. 4). In the absence of 6-azauracil, PieF expression resulted in a modest growth inhibition, which was similar, although not as severe, as what we observed for the *POP2* deletion strain (Fig. 4A). Like a *POP2* deletion, expression of PieF sensitized yeast to the drug 6-azauracil, resulting in a severe growth defect (Fig. 4B). As above, we used the unrelated effector Lpg2149 as a negative control. Taken together, PieF expression phenocopies the deletion of *POP2* in *S. cerevisiae*, suggesting that PieF can impinge on CCR4−NOT activity within the eukaryotic cell.

### Heterologous expression of PieF in mammalian cells results in pleiotropic effects

While PieF inhibits CNOT7 deadenylase activity *in vitro* and phenocopies the genetic ablation of this activity in yeast*,* some endogenous modulators of the CCR4−NOT complex with inhibitory roles *in vitro* end up being more nuanced when examined in live cells. An example is BTG2, which inhibits CNOT7 deadenylase activity in a dose-dependent manner *in vitro* (97, 104)*,* but in the cellular context directs the complex to degrade mRNA transcripts required for cellular proliferation (74, 75, 87, 96, 105–109). Like PieF, BTG1/2 are small proteins with no catalytic core or defined RNA-binding domains (97). As *L. pneumophila* is known to possess effector proteins that mimic the functions of eukaryotic proteins (28, 29, 110), we sought to further compare the CCR4−NOT modulatory activities of PieF and BTG/TOB proteins.

An established method for studying CCR4−NOT activity within the cellular context is the artificial tethering of proteins to mRNA reporter transcripts (111). When tethered, components of the CCR4−NOT complex and BTG/TOB proteins destabilize reporter mRNA and reduce reporter gene expression in live cells (96, 112, 113). BTG/TOB-mediated destabilization is due to the recruitment of endogenous CCR4−NOT complex (72–74, 96). The *in vitro* (97, 104) and *in vivo* (74) discrepancies of BTG/TOB activity prompted us to assess the impact of PieF on RNA stability and expression. To this end, we leveraged the bacteriophage λN-BoxB tethering assay in HEK293T cells (114–117). Using this approach, we measured the impact of tethering CNOT7, PieF, or BTG1 to two different reporters: i) a standard *Renilla* reporter with five BoxB hairpins within its 3′ UTR or ii) a modified *Renilla* reporter with five BoxB hairpins within its 3′ UTR as well as a self-cleaving hammerhead ribozyme (HhR), which generates a protected poly(A) tail that is not accessible to 3′−5′ exonucleases (116)—the HhR-modified reporter allows for deadenylation-independent mechanisms of silencing to be assayed.

**Fig 5:**
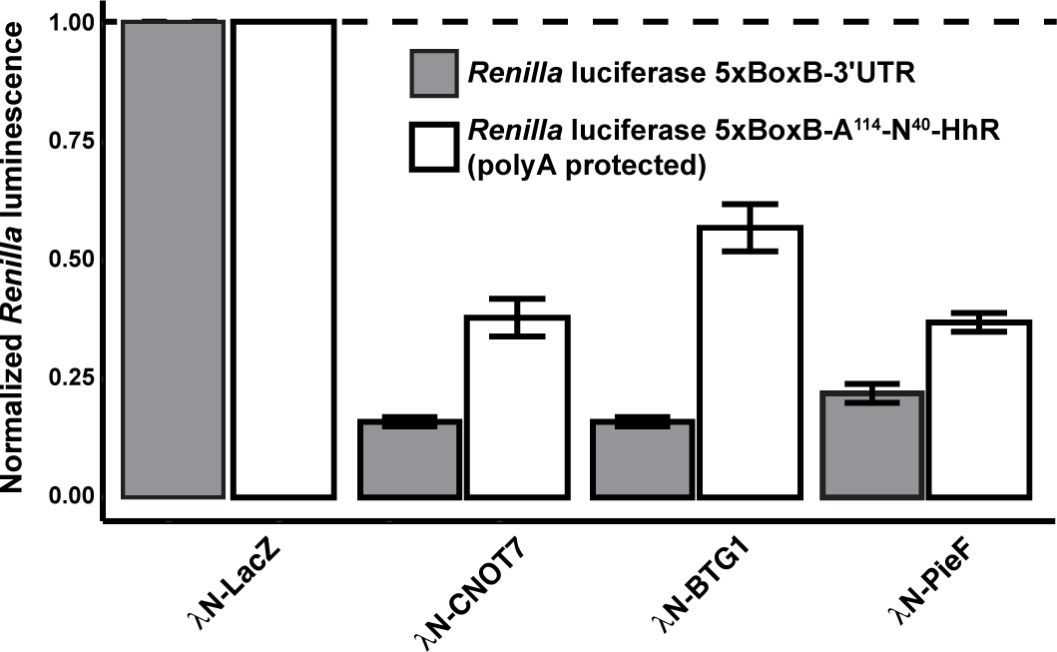
Tethering of PieF to an mRNA reporter results in deadenylase-dependent and -independent silencing. Using a *Renilla* luciferase reporter construct with five BoxB hairpins within the 3’ UTR (*“Renilla* luciferase 5xBoxB-3′UTR”—grey boxes), *Renilla* luciferase activity is measured relative to a firefly luciferase control. A modified, luciferase reporter construct with 5xBoxB hairpins and a protected poly(A) tail (*“Renilla* luciferase 5xBoxB-A114-N40-HhR”—white boxes) serves as a control to assess the requirement of deadenylation on silencing. The *Renilla* luciferase activity is normalized to firefly luciferase activity for each sample, and the ratio is plotted relative to a λN-LacZ control. Activity data represent an average and standard deviation of three biological replicates. Student’s *t*-test was calculated on the ratio of *Renilla*/firefly luminescence to determine significance, with all values *P* < 0.01 relative to the LacZ control).

Under each condition, the results with PieF, BTG1, and CNOT7 were comparable: all three proteins reduced reporter expression (Fig. 5, gray boxes). This effect was not observed for the standard negative control LacZ. Notably, for PieF, CNOT7, and BTG1 (but not LacZ), we also observed reduced expression of the polyA-protected (HhR) luciferase reporter construct (Fig. 5, white boxes), consistent with deadenylation-independent mechanisms of reporter silencing. Taken together, we interpret these results to suggest that PieF can recruit the CCR4−NOT complex to mRNA substrates, likely resulting in a combination of deadenylase-dependent and deadenylase-independent silencing. While this is not unexpected for artificial tethering assays, it may also reflect multiple functional outputs of the PieF−CNOT7 interaction *in vivo*. Indeed, subunits of the CCR4−NOT complex, including catalytically active and inactive CNOT7, have illustrated translational silencing independent of deadenylation activity (118–120). The GW182 protein family (TNRC6A, B, C) of the miRNA silencing pathway also translationally represses targets through CCR4−NOT-mediated PABP dissociation that is not dependent on deadenylase activity (116).

### Overexpression of PieF in mammalian cells results in the nuclear exclusion of CNOT7

**Fig 6:**
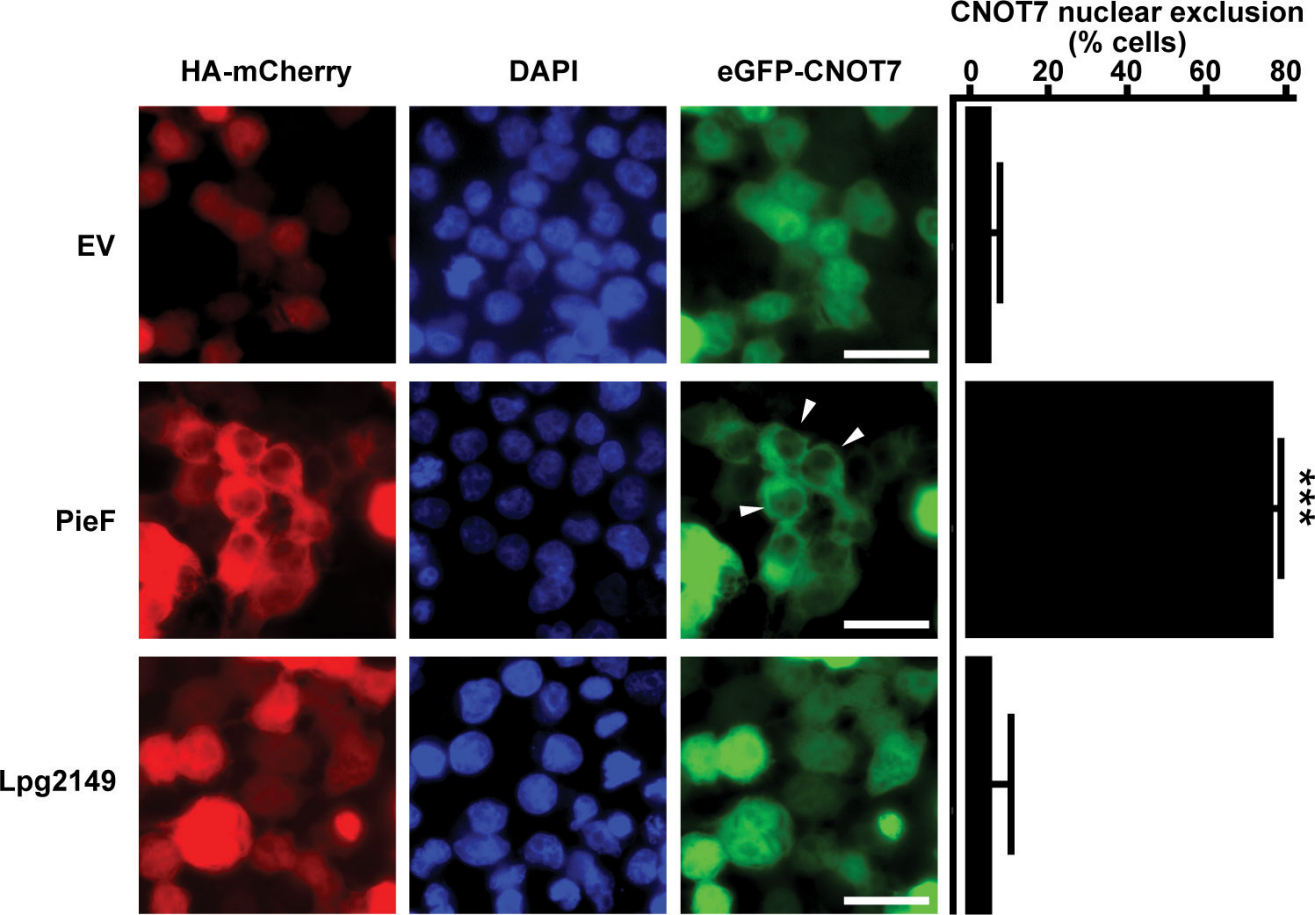
PieF restricts CNOT7 nuclear localization following ectopic expression in HEK293T cells. Transient transfection of HEK293T cells with eGFP-CNOT7 and HA-mCherry PieF, Lpg2149, or an empty vector control causes a nuclear exclusion phenotype specific to PieF and visible under a 40× oil immersion objective. The scale bar represents 50 µm. Arrows illustrate representative nuclear exclusion phenotypes. 4',6-Diamidino-2-phenylindole (DAPI) illustrates nuclear staining. Images are representative of at least two biological replicates. Manual quantification of cells (>100 per replicate) with nuclear-excluded eGFP-CNOT7 shows a statistically significant difference in the PieF populations relative to other treatments using a Student’s *t*-test (*** =*P* < .01) across two biological replicates.

CNOT7 displays a dynamic localization pattern, shuttling between cellular compartments during the cell cycle (121). In the context of host remodeling, changing CNOT7 localization would be one way to further modify its activity and/or the pool of mRNA transcripts that it can silence. We therefore assayed whether PieF expression influences the localization of CNOT7 following ectopic expression in HEK293T cells. CNOT7 was N-terminally tagged with eGFP and co-transfected with HA-mCherry PieF or HA-mCherry Lpg2149 (an unrelated negative control). After transfection, most cells in the HA-mCherry vector or HA-mCherry Lpg2149 treatment demonstrated diffuse pan-cellular localization of eGFP-CNOT7 (Fig. 6), consistent with other published CNOT7 localization data (122). In contrast, HA-mCherry PieF expression resulted in a significant proportion of the cell population showing a diminished eGFP signal within the nucleus (Fig. 6). Taken together, our data demonstrate that PieF, in addition to its other modulatory activities described above, redirects the DEDD nuclease CNOT7 from the nucleus.

### PieF modulates CCR4−NOT subunit composition *in vivo*

**Fig 7:**
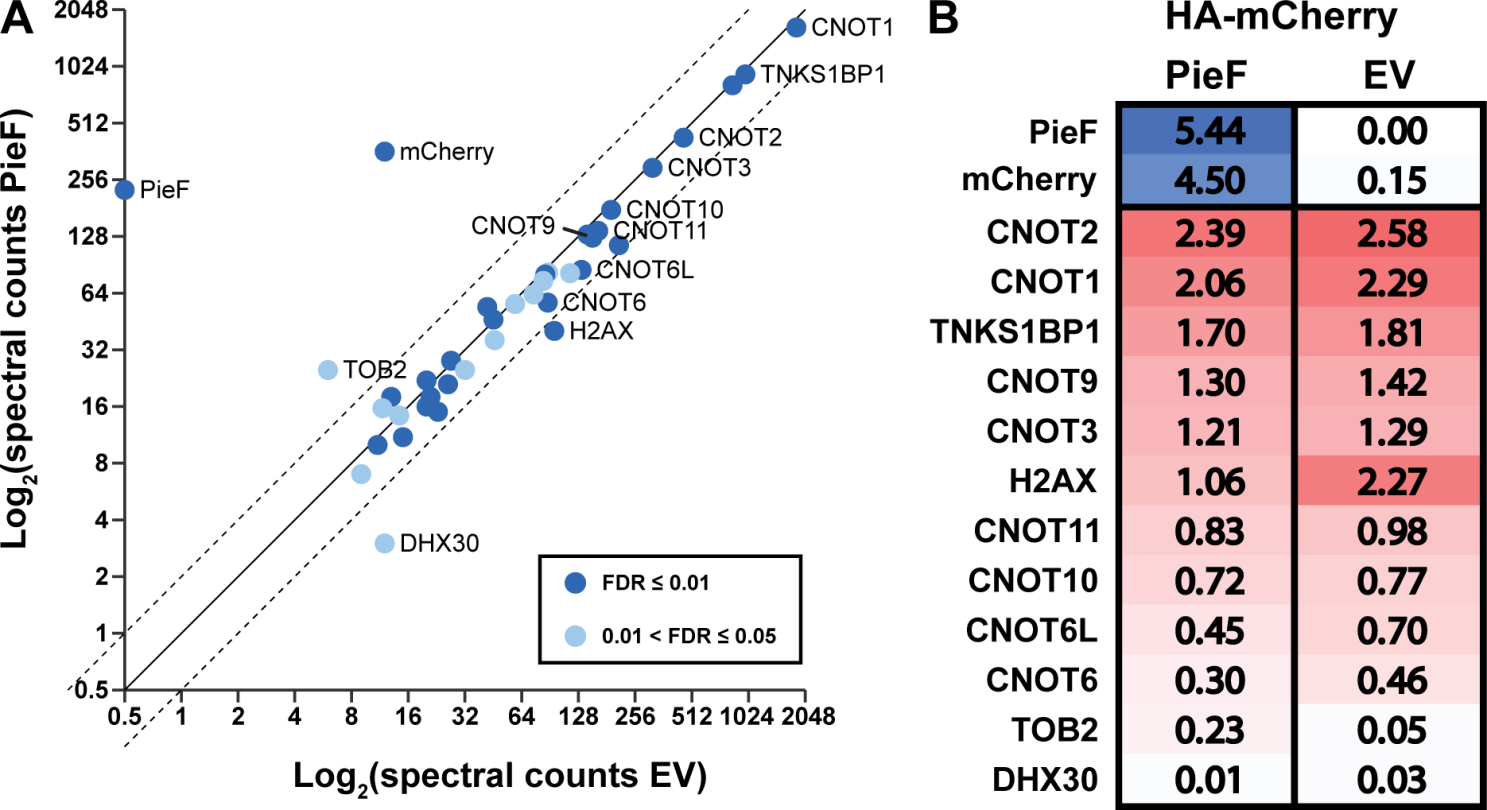
PieF modulates CCR4−NOT complex composition. A stable Flp-In™ T-REx™ 293 cell line with FLAG-tagged CNOT7 was transfected with HA-mCherry-PieF or an empty vector control and subjected to FLAG IP-MS to assess subunit composition changes in CCR4−NOT. (**A**) A spectral abundance scatter plot depicting SAINT analysis of IP-MS results. The plot illustrates the depletion of the EEP nuclease module (CNOT6/6L) relative to the empty vector control. Canonical CCR4−NOT components and TNKS1BP1 are labeled, as well as proteins that exceed the twofold change thresholds (dashed lines). (**B**) A heatmap representation for average spectral counts normalized to protein size between PieF and EV treatment.

To determine how PieF expression influences CCR4−NOT subunit composition within mammalian cells, we immunoprecipitated FLAG-tagged CNOT7 from HEK293T cells co-expressing PieF or an empty vector control. While this approach is designed to enrich for all CNOT7 complexes in the cell, some of which are PieF-bound, some of which are likely not, we nevertheless observed a decrease of 36% of CNOT6 and 35% of CNOT6L spectral counts co-precipitated by CNOT7 upon PieF co-expression relative to an empty vector control (Fig. 7A and B). Notably, other components of CCR4−NOT did not show such marked changes in abundance between treatment conditions, suggesting that the rest of the canonical CCR4−NOT complex remains largely intact. CNOT11 was the next most differentially enriched, with 84% of total peptides being captured in the PieF co-precipitation relative to empty vector. Consistent with the nuclear exclusion phenotype that we observe for CNOT7 in mammalian cells, expression of PieF in this assay also resulted in a marked reduction of the nuclear localized histone H2AX and an increase in the cytoplasmic localized TOB2 in the CCR4−NOT complex (123) (https://www.proteinatlas.org/). Taken together, these results suggest that the *in vivo* activity of PieF involves several conserved host proteins with the potential to profoundly impact the translational program of host cells.

### Yeast two-hybrid analysis of PieF−CNOT7, PieF-CDK4, and CNOT7-BTG1 suggests distinct interaction interfaces and additional regulatory complexity

**Fig 8:**
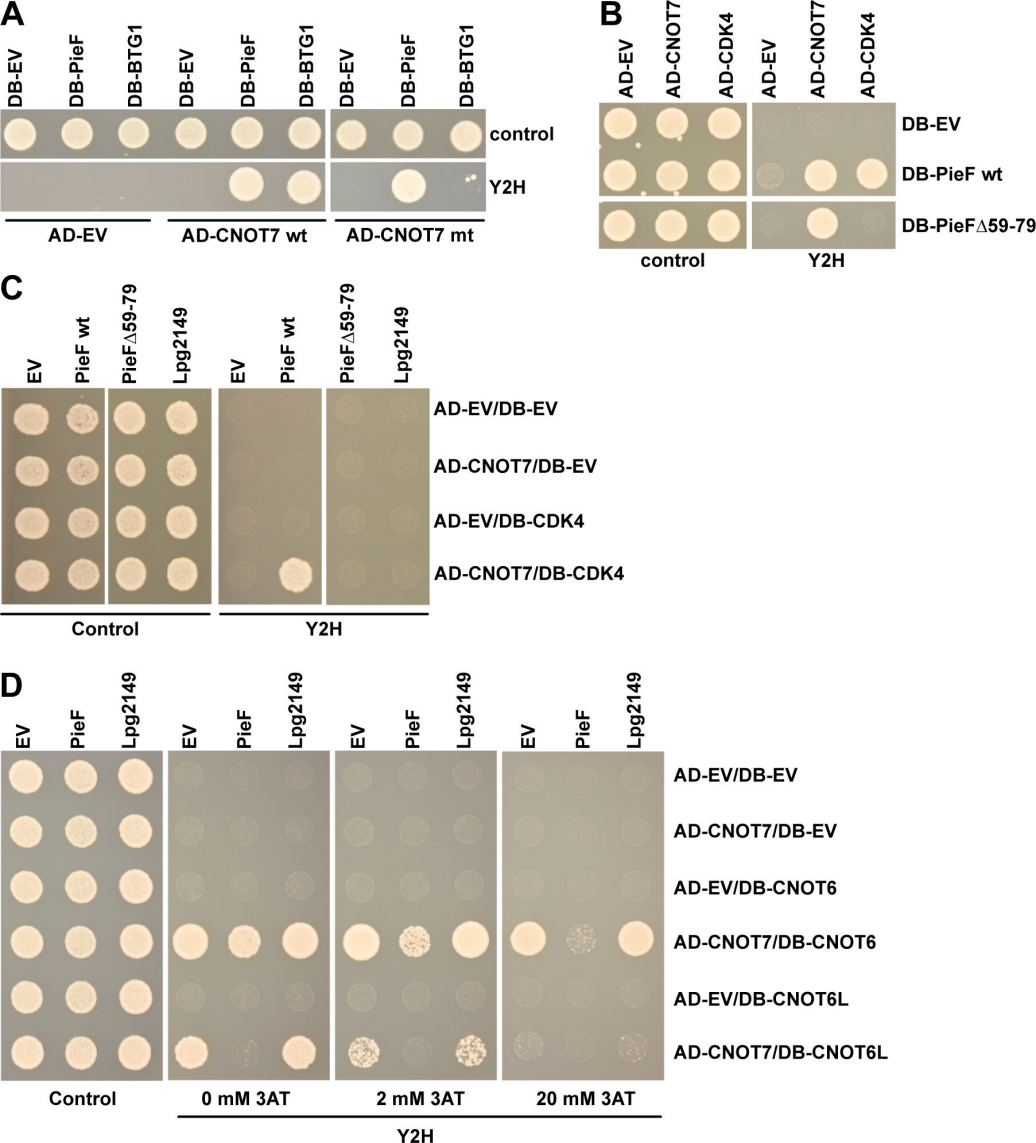
Differential PieF binding interfaces with CNOT7 and CDK4. (**A**) Y2H assay with DB-PieF and wild-type or mutant AD-CNOT7. Yeast strains carrying DB- and AD-fusions or empty vector controls were diluted to an OD_600_ = 1 and spotted on control medium (SC-Leu/Trp) or Y2H selective medium (SC-Leu/Trp/His) and grown 2 days before imaging. The CNOT7 E247A, Y260A mutant was previously shown unable to interact with BTG2 (124). The assay was performed in triplicate, and a representative example is shown. (**B**) Y2H assay with the PieFΔ59–79 mutant (deletion of a putative predicted coiled-coil region). DB-PieFΔ59–79 was still able to interact with CNOT7, but not CDK4. The assay was performed as above and in triplicate with a representative example shown. (**C**) A Y2H assay between AD-CNOT7−DB-CDK4 with a constitutive expressed third protein (PieF, Lpg2149, or an EV control). Yeast strains carrying three vectors were spotted onto control medium (SC-Ura/Leu/Trp) or Y2H selective medium (SC-Ura/Leu/ Trp/His) and grown 2 days before imaging. The AD-CNOT7−DB-CDK4 combination with EV or Lpg2149 cannot grow on Y2H selective medium. Co-expression of PieF allows growth on Y2H selective medium, while co-expression of PieFΔ59–79 does not. The assay was performed in triplicate, and a representative example is shown. (**D**) A Y2H assay on the AD-CNOT7−DB-CNOT6 and DB-CNOT6L pair with a constitutively expressed third protein was performed as described above, with addition of 2 and 20 mM 3AT (an inhibitor of the HIS3 reporter product) to increase stringency of the Y2H selection. Co-expression of PieF, but not Lpg2149 or EV control, disrupts the Y2H signal between CNOT6 and CNOT6L. The assay was performed in triplicate, and a representative example is shown.

With published structural insight into the interaction between CNOT7 and other eukaryotic factors (such as BTG/TOB and CNOT6/6L) and validated interactions between PieF and at least two different host factors (CNOT7/8 and CDK4), we next sought to determine the relative genetic constraints of each of these interactions. Through examination of co-crystal structures, CNOT7 residues required for BTG/TOB interaction (E247, Y260) have been identified and experimentally validated (124). To test the impact of these mutations on PieF binding, we performed the Y2H assay between PieF and either wild-type CNOT7 or CNOT7^E247A,Y260A^. As expected, mutating both residues in CNOT7 abrogated its interaction with BTG1; however, these same mutations failed to disrupt the CNOT7–PieF interaction (Fig. 8A). Notably, this suggests different CNOT7 binding constraints for PieF and BTG/TOB proteins.

We next sought to explore the relationship between our observed interactions between PieF−CNOT7 and PieF−CDK4 (Fig. 1B). While PieF is largely devoid of predicted secondary structure, deletion of a predicted coiled-coil region in PieF (residues 59–79) did not affect the interaction of PieF with CNOT7, but instead abrogated the Y2H interaction of PieF with CDK4 (Fig. 8B). This suggests that the interaction interfaces on PieF with CNOT7 and CDK4 are separate and distinct.

Given the genetic separability of the PieF−CNOT7 and PieF−CDK4 interactions, we next sought to determine whether PieF can interact with CNOT7 and CDK4 concomitantly. We expressed PieF or a negative control (Lpg2149) in a Y2H strain carrying vectors expressing AD-CNOT7 and DB-CDK4. Alone (EV) or with Lpg2149, the AD-CNOT7–DB-CDK4 pair does not have a Y2H interaction (Fig. 8C). However, when PieF is co-expressed, AD-CNOT7−DB-CDK4 can grow under Y2H selective conditions, and this abrogated when co-expressing the PieF∆59–79 mutant (Fig. 8C). Taken together, PieF appears to have separate and distinct interaction sites for CNOT7 and CDK4 and can form a co-complex with CNOT7 and CDK4, adding an additional layer to its regulatory complexity *in vivo*. One intriguing possibility is that CDK4 or other related kinases may influence the regulation of CNOT7 by PieF once inside the host cell.

Finally, we examined the effect of PieF on the interaction between CNOT7 and the EEP-like deadenylase CNOT6/6L. We performed a Y2H assay between CNOT7 and CNOT6/6L in the presence of a third untagged protein (PieF or control Lpg2149) that was constitutively expressed from a low-copy vector. Expression of PieF, but not Lpg2149, was sufficient to diminish the Y2H signal between CNOT7 and CNOT6 and CNOT6L (Fig. 8D). Notably, this is consistent with the PieF-induced reduction of spectral counts for CNOT6 and CNOT6L in our mass spectrometry analysis of CCR4−NOT complexes above (Fig. 7).

## DISCUSSION

Within the *pie* locus (30), PieA, PieE, and PieG appear to influence vesicular trafficking and LCV motility (3, 31–35), whereas PieF appears to instead target distinct eukaryotic biology−mRNA stability. While several effectors are known to redundantly target protein expression, our characterization of PieF activity suggests the existence of a complementary, upstream mechanism by which *L. pneumophila* can further interfere with host expression. To our knowledge, PieF is the first described virulence protein encoded by a human bacterial pathogen that intersects the eukaryotic CCR4−NOT deadenylase machinery.

In the literature, there is a prominent example of another pathogenic factor that targets the CNOT7 subunit of CCR4−NOT (92). The *Xanthomonas citri* effector, PthA4, inhibits *Citrus sinensis* CNOT7 activity *in vitro* and promotes transcription and translation of factors that facilitate *X. citri* canker formation (92). This is one of several examples of the CCR4−NOT complex providing protective effects against environmental stressors and pathogens in plants (92, 122, 125, 126). There are also studies demonstrating that CNOT7 is required for the development of gametocytes in the malarial parasite *Plasmodium falciparum* (127), and CNOT7 orthologs also play a role in diverse stress response phenotypes within model and pathogenic fungi alike (128).

The CCR4−NOT complex is found across eukaryotes (60), making it an attractive target for a bacterial pathogen like *L. pneumophila* that can replicate in host cells as evolutionarily diverse as protists and human macrophages (3, 4). Despite having conserved CCR4−NOT components, the protozoan CCR4−NOT complex is largely unstudied (129, 130). CCR4−NOT components (including CNOT7) are found in protists, such as *Entamoeba histolytica* (129, 130). Furthermore, in some trypanosome species, CNOT7 is the only conserved deadenylase and essential for growth (129–133). The DEDD nucleases (CNOT7/8) are considered the primordial nuclease module of CCR4−NOT, being much more conserved throughout eukaryotic evolution than the EEP nuclease module (133). CNOT7/8 is much more likely to be conserved across evolutionarily diverse eukaryotic hosts and represents a prime pathogenic target. Indeed, our observation that PieF expression perturbs CNOT7 activity in *S. cerevisiae* supports a role for this effector in modulating CCR4−NOT activity across the evolutionarily diverse protozoan hosts of *L. pneumophila*.

As a central regulator of eukaryotic gene expression with roles in immune signaling (134) and stress response pathways (128), there are several ways in which modulation of the CCR4−NOT complex by PieF could influence host cell gene expression, ranging from generalized changes to mRNA stability to the modulation of specific targets. Given this, pinpointing which of these downstream effects specifically contribute to *L. pneumophila* pathogenesis is likely to be a challenge. Our observation of PieF-directed CNOT7 localization changes is perhaps informative in this regard, suggesting an upstream influence on CNOT7 activity that may nevertheless modulate specific classes of transcripts based on one or more general characteristics. Given our observation that the *in vitro* inhibition of CNOT7 by PieF is stochiometric (Fig. 3B), the interpretation of any phenotypes ascribed to PieF overexpression will ultimately need to take into account that the endogenous levels of PieF compared with CNOT7 are likely to be highly sub-stochiometric during infection.

In the environment, resources are often scarce, and many protozoan cells are in stationary phase (135). Endogenous CNOT7 is almost exclusively nuclear in starved mammalian MRC5 cells (121), and this may best approximate the expected configuration of amoebal CCR4−NOT in a nutrient-poor environment as well. Notably, in mammalian cells overexpressing PieF, we observe a striking cytoplasmic relocalization of CNOT7. During *L. pneumophila* infection of one or more protozoan hosts, PieF may re-direct nuclear CNOT7 activity towards the cytoplasm, which might ultimately favor bacterial replication by destabilizing specific mRNA subsets, blocking the host cell cycle, or facilitating translational repression through CCR4−NOT recruitment. *L. pneumophila* has been previously shown to actively arrest the host cell cycle in both mammalian, and protozoan cells through the activity of numerous effectors (54, 135–137), aiding the formation of a replication permissive compartment. During infection, it is important for the bacterium to block host entry into S-phase, as this cell cycle progression destabilizes the LCV and prevents bacterial replication (135). One attractive model is that PieF contributes to this block. If so, in addition to examining the role of PieF in post-mitotic macrophages, it will be important to look for specific effects on cell cycle progression and bacterial replication in one or more carefully chosen natural hosts.

In the future, it will be important to delineate which mRNA transcripts are influenced by PieF both ectopically and during infection. Bulk RNA sequencing following transfection of PieF might reveal specific transcripts that are sensitive to its modulation of CNOT7. Additionally, global changes to poly(A) tail length could be monitored using a TAIL-seq-based approach (138) to see whether PieF can trigger general mRNA deadenylation (87). If the role of PieF is to block host cell-cycle progression, the target transcripts may largely overlap with those of the BTG/TOB family (87). Such a role would explain why no replication defect is observed for a *pieF* mutant in terminally differentiated, post-mitotic macrophages (25, 26).

Despite the experimental challenges presented by the breadth of biology that it intersects, the highly conserved CCR4−NOT complex presents an enticing and unexplored avenue for modulating the host from a pathogenic perspective (106, 107, 109).

## MATERIALS AND METHODS

### Bacterial strains and plasmids

All plasmids used and generated in this study are listed in Table S2, and the primers used are listed in Table S3. *E. coli* strain Top10 and XL1blue (Invitrogen) were used for routine cloning and plasmid maintenance; DB3.1 (Invitrogen) was used to maintain empty Gateway vectors, BL21-GOLD (DE3) (Stratagene) was used for protein expression and purification. The *E. coli* strain BTH101 (Euromedex) [*F^0^ cya-99 araD139 galE15 galK16 rpsL1 (Str^R^) hsdR2 mcrA1 mcrB1 relA1*] was used for the bacterial adenylate cyclase two-hybrid (BACTH) assay. *S. cerevisiae* toolbox strains RY1010 (MATa *leu2-3,112 trp1-901 his3-200 ura3-52 gal4∆ gal80∆ PGAL2-ADE2 LYS2::PGAL1-HIS3 MET2::PGAL7-lacZ cyh2^R^ can1∆::PCMV-rtTA-KanMX4*) and RY1030 (*MATα leu2-3,112 trp1-901 his3-200 ura3-52 gal4∆ gal80∆ PGAL2-ADE2 LYS2::PGAL1-HIS3 MET2::PGAL7-lacZ cyh2^R^ can1∆::TADH1-PtetO2-Cre-TCYC1-KanMX4*) (139) were used for yeast two-hybrid (Y2H) assays and were grown on YPD medium supplemented with 180 mg/L adenine sulfate or on synthetic defined medium (SC) lacking amino acids as indicated and with 180 mg/L adenine sulfate and 2% glucose. *S. cerevisiae* strains BY4741 (*MATa his3∆1 leu2∆0 lys2∆0 ura3∆0* [140]) and BY4741 *pop2∆::KanMX4* (141) were used for growth curve assays and were grown on YPD medium or on SC medium lacking uracil with 2% glucose (SC-Ura).

The *Legionella pieF* and human CNOT open reading frames were PCR amplified from Lp02 genomic DNA or plasmid templates indicated in Table S2, respectively. For the bacterial adenycyclate two-hybrid (BACTH) assay, primers were designed using NEBuilder software (V2.4.0), and the PCR products were incubated with XbaI linearized pKT25 and pUT18C (83) and NEBuilder HiFi DNA assembly cloning mix (New England Biolabs) according to the manufacturer’s instructions and transformed to XL1-blue. Similarly, the pCI-λN-HA-CNOT7, BTG1, and PieF vectors for the tethering assay were cloned using NEBuilder designed primers. PCR products were cloned into pCI-λN-HA digested with EcoRI/NotI using NEBuilder HiFi DNA assembly cloning mix. For protein purification, *pieF* and CNOT7 were PCR amplified and cloned into pMCSG68-SBP-TEV (*pieF*) or pMCSG53 (CNOT7) by LIC-independent cloning (142). To clone pDONR221CNOT6stop, CNOT6 was PCR amplified from pT7-EGFP-C1-HsNot6 (Addgene plasmid # 37368; a gift from Elisa Izaurralde) (143) and cloned into pDONR221 (Invitrogen) using BP clonase II (Life Technologies) according to manufacturer’s instructions. To clone pDONR221MAP2K3, MAP2K3 was PCR amplified from pMT2-HA-MKK3 (Addgene plasmid # 21580; a gift from John Kyriakis) (144). pDONR221-pieF∆59–79 and pDONR221CNOT7 Y260A E247A were made using the Q5 site-directed mutagenesis kit (New England Biolabs). For CNOT7, the mutations were introduced sequentially. All plasmids were sequence verified. pDONR entry clones were cloned into Gateway destination vectors pDEST-DB-ccdB, pDEST-AD-ccdB (Y2H assays) (145), pDEST53-HAmCherry (microscopy), pcDNA5′3XFLAG-ccdB (146) (IP-MS assay) or pAG416GPD-ccdB (Addgene plasmid # 14148, a gift from Susan Lindquist) (147) (Y2H and growth curve assays) using LR clonase II (Life Technologies) according to manufacturer’s instructions.

### Protein purifications

Protein purifications were performed according to the QIAexpressionist protocol and as previously described (148). Briefly, *E. coli* BL21-Gold (DE3) chemically competent cells were transformed with either pMCSG68-SBP-PieF or pMCSG53-CNOT7 and grown in 1 L of LB broth with 100 µg/mL carbenicillin. Cultures were grown to an OD_600nm_ of 0.6 at 37°C, and expression was induced with 1 mM isopropylbeta-D-thiogalactopyranoside (IPTG) for 5 h at 37°C. Cells were harvested by centrifugation (12,000×*g* for 10 min at 4°C). After resuspension in 40 mL lysis buffer (300 mM NaCl, 5% glycerol, 5 mM imidazole, 50 mM HEPES pH 7.5), cells were lysed by sonication (30% amplitude, 10 s on, 10 s off for 10 min) in the presence of 1 mM phenylmethylsulfonyl fluoride (PMSF). The lysates were clarified by centrifugation (35,000×*g* for 20 min at 4°C), and the soluble fractions were incubated with 2 mL Ni-NTA resin (Qiagen). Columns were washed with 300 mL wash buffer (300 mM NaCl, 30 mM imidazole, 15 mM HEPES pH 7.5, 5% glycerol) and eluted in 40 mL of elution buffer (300 mM NaCl, 300 mM imidazole, 15 mM HEPES pH 7.5). Eluates were dialyzed, quantified, and concentrated in dialysis buffer to >5 mg/mL (15 mM HEPES pH 7.5, 300 mM NaCl, 1 mM dithiothreitol [DTT]) using Vivaspin 10 kDa cutoff columns (GE Healthcare). The 6xHis-tag was cleaved from CNOT7 for biolayer interferometry by incubating the protein with 6xHis-tagged TEV protease (60 µg/mg) overnight at 4°C. TEV protease and uncleaved protein were then removed by nickel resin purification, with the eluate kept for downstream assays. Purified Lpg2149 protein was a kind gift from Drs. D. Valleau and A. Savchenko, with detailed methods described in reference 149.

### Identification of PieF host-interacting proteins by AP-MS

To identify putative effector–host target interactions, we used the affinity purification coupled with mass spectrometry (AP-MS) approach as described (77, 78) for two individual technical replicates. Purified 6xHis-SBP PieF was incubated with magnetic streptavidin beads (Life Technologies) for 1 h in affinity purification buffer (50 mM HEPES pH 8, 150 mM NaCl, 1 mM EDTA, 0.2% NP-40). Human cell lysate from 5 × 10^7^ U937 cells was prepared through freeze-fracture lysis in affinity purification buffer with protease inhibitor cocktail (MilliporeSigma, P8340). The lysate was pre-cleared with streptavidin-conjugated beads to remove biotin-conjugated proteins and further clarified through centrifugation (14,000 × *g* for 15 min at 4°C). Streptavidin-bound PieF was then incubated with the clarified cell lysate for 3 h at 4°C, purified through magnetic separation, and resuspended in 50 mM ammonium bicarbonate pH 8.0. Proteins were eluted by incubating with 2.5 mM biotin for 15 min and digested overnight at 37°C with sequencing grade-modified trypsin (Promega). Trypsin digestion was terminated through the addition of trichloroacetic acid (0.5% final). Peptides were purified using C18 OMIX tips (Agilent) and eluted from the C18 tips using 0.1% formic acid in acetonitrile and dried by vacuum centrifugation. Before mass spectrometry, the peptides were resuspended in 0.1% formic acid in water, centrifuged, and loaded in technical duplicate using an EASY-nLC II autosampler onto a 10 cm C18 column with a glass nano-spray ionization tip using EASY-nLC II liquid chromatography system (Thermo Scientific). For data collection, a Q-Exactive spectrometer was used in positive ion mode for a 120 min gradient on increasing acetonitrile concentration. Raw data files were converted using MSconvert and queried against the human and *L. pneumophila* proteome collection using GPM/X! tandem (http://www.thegpm.org/). Data were then analyzed using ProHits (150) by comparing the experiment to 20 unrelated AP-MS experiments with 11 unique baits as background controls. Filtering metrics included a GPM expect score of >−10, and <8 unique peptides. The ribosomal, keratin, and artifact protein biofilters were also applied and excluded prey are listed in Table S4. Data were sorted by unique peptides identified and normalized to protein size in kDa.

### Yeast two-hybrid assays

Yeast two-hybrid (Y2H) experiments were performed similarly to those described (151). Bait and prey proteins were fused N-terminally to the DNA-binding and transcriptional activation domains of the Gal4p transcription factor, respectively. pDEST-AD vectors were transformed to yeast strain RY1010, while pDEST-DB vectors were transformed to RY1030 using the LiAc/SS-DNA/PEG method (152). Transformants were selected on agar plates with synthetic defined medium lacking tryptophan (SC-Trp, pDEST-AD vectors) or medium lacking leucine (SC-Leu, pDEST-DB vectors) for 2 days at 30°C. The resulting haploid strains of opposite mating type were mated on YPD agar, incubated for 24 h at 30°C, struck onto SC-Leu/Trp agar plates and incubated for 2 days at 30°C to select for diploid strains carrying both vectors. To query for physical interactions between AD- and DB-fusions, diploid strains were grown overnight in SC-Leu/Trp medium, diluted to an OD_600_ of 1 and stamped onto Y2H-selective medium lacking leucine, tryptophan and histidine (SC-Leu/Trp/His) using the VP 407AH pin tool (V&P Scientific). Plates were incubated at 30°C for 2 d and imaged.

Y2H experiments with a constitutive expressed third protein were performed with a third vector pAG416-GPD-ccdB, which contains a Ura auxotrophic marker and constitutive GPD promoter (84). Diploid strains carrying AD and DB-fusion vectors were transformed with pAG416GPD-PieF, Lpg2149, and an empty vector control. Transformants were selected on agar plates with medium lacking uracil, leucine, and tryptophan (SC-Leu/Trp/Ura) and grown for 2 days at 30°C. To query for physical interactions or the loss of physical interactions between AD- and DB-fusions, the diploid strains carrying three plasmids were grown overnight in 1 mL SC-Leu/Trp/Ura, normalized to 1 OD_600_ and spotted onto control medium (SC-Ura/Leu/Trp) or Y2H-selective media (SC-Ura/Leu/Trp/His) supplemented with 0, 2, or 20 mM 3AT, an inhibitor of the reporter gene *HIS3* product.

Y2H screens of pDESTAD-CNOT6, −6L, −7, –8, and −9 fusions against a DB-effector fusion array were performed as follows. Copper-inducible pNZM1100Cup1-DB effector plasmids (85) were transformed to RY1030 using a 96-well LiAc/SS-DNA/PEG transformation protocol (153) and selected on SC-Leu agar plates prepared with yeast nitrogen base (YNB) lacking copper (ForMedium). Two colonies per DB-fusion were arrayed in 384-format using an S&*P* BM5 robot (S&*P* Robotics Inc), creating three DB-array plates with a total of 348 inducible DB-effector fusions and converted to a 1,536-format, creating quadruplicate spots for each strain. Each haploid query strain (RY1010+pDESTAD vector) was mated individually to each strain on the DB-effector array as described (78) using the S&*P* BM5 robot with minor modifications. Query strains (RY1010 carrying AD-CNOT vectors or an empty vector) were grown as a lawn on SD-Trp agar OmniTray plates (Thermo Scientific). To mate the AD query strains with the DB array strains, the haploid strains were pinned on top of each other on YPD agar plates, incubated overnight at 30°C and subsequently pinned onto SC-Leu/Trp agar plates lacking copper to select for diploid strains carrying both plasmids. Diploid strains were pinned onto SC-Leu/Trp agar plates without copper or SC-Leu/Trp/His agar plates with 1 mM CuSO_4_ to induce expression of the DB-effector fusions. Plates were grown 2 days at 30°C before imaging. Spot sizes were analyzed using SGAtools (http://sgatools.ccbr.utoronto.ca/; Wagih et al., 2013) and analyzed as described (78). The 348 effectors represented on the array and normalized spot sizes can be found in Table S5.

### BACTH assay

The BACTH assay was performed as described previously (82, 83) with minor modifications. Briefly, *E. coli* strain BTH101 was made chemically competent by incubating with ice-cold 50 mM CaCl_2_, co-transformed with plasmids pKT25 and pUT18C, recovered in 1 mL LB broth and selected on LB plates supplemented with 50 µg/mL kanamycin, 100 µg/mL carbenicillin, 50 μg/mL streptomycin, and 0.5 mM IPTG at 37°C. For each combination, three individual co-transformant clones were grown overnight at 37°C in 1 mL LB supplemented with antibiotics and 0.5 mM IPTG as above, diluted 10 times and spotted onto LB agar with 50 µg/ml 5-bromo-4-chloro-3-indolyl-β-D-galactopyranoside (X-gal) or MacConkey agar with 1% (wt/vol) maltose, both supplemented with 0.5 mM IPTG and antibiotics as above using the V&P 407 AH tool (V&P Scientific). Plates were incubated overnight 30°C and imaged.

### Biolayer interferometry binding assays

Biolayer interferometry binding assays were performed on the Octet RED96 platform (PALL-ForteBio). Sensor-bound samples were diluted to 5 µg/mL in kinetics buffer for each assay (1× PBS, 0.1  mg/mL bovine serum albumin, 0.002% Tween 20) for each assay. Streptavidin biosensors were equilibrated in kinetics buffer and then moved to purified tagged PieF solution at 5 µg/mL or kinetics buffer alone. Following a 200 s loading period, the sensors were again washed for 450 s in kinetics buffer and incubated with a range of concentrations of untagged catalytically active CNOT7 (0, 27, 54, 108, 270, 540, 1,080 nM). Following an association period of 300 s, the sensors were moved to kinetics buffer for 450 s to allow dissociation of CNOT7. Data analysis was performed using the GraphPad Prism software (Prism 9). Data were fitted through non-linear regression using a one-site-specific binding model. Each measurement was performed a minimum of three times in independent experiments.

### Deadenylation assay

Purified proteins 6xHis-SBP-PieF, 6xHis-CNOT7, and 6xHis-SBP-Lpg2149 (a gift from Dr. Dylan Valleau, purified as described [149]), were diluted in 25 µL deadenylase buffer (50 mM Tris-HCl pH7.5, 10% glycerol, 1 mM DTT, 2 mM MgCl_2_, 150 mM NaCl). Combinations of diluted 6xHis-CNOT7 with 6xHis-SBP-PieF, 6xHis-SBP-Lpg2149 or deadenylase buffer were pre-incubated at room temperature (RT) in 50 µL reactions. The final concentration of 6xHis-CNOT7 or 6xHis-SBP-Lpg2149 was 1 µM, while 6xHis-SBP-PieF was 1, 0.5, or 0.25 µM as determined by NanoDrop Spectrophotometer (Thermo Scientific). Each of the purified proteins was also tested alone in the deadenylation assay at 1 µM. Following pre-incubation, 2.5 µL of PAGE-purified 10 µM 5′−6 carboxyfluorescein-labeled RNA substrate with a poly(A) tail of 20 nucleotides [5′-FAM-UCUAAAUAAAAAAAAAAAAAAAAAAAA-3′] (IDT) was added to each reaction and incubated at 37°C for 30 min. Reactions were stopped by the addition of RNA Gel Loading Buffer II (Thermo Fisher Scientific) and boiling at 95°C for 5 min. Reactions (5 µL) were resolved on an 8M UREA 25% polyacrylamide gel, fixed in 1× TBE with 10% acetic acid and 10% methanol for 5 min at RT and imaged on a Typhoon FLA 9500 (GE Healthcare) at 800 V using a 488 nm excitation wavelength. The gel image is representative of at least three independent experiments.

### λN tethering assay

HEK293T cells were seeded in 24-well plates at 2 × 10^5^ cells per well with Dulbecco’s Modified Eagle Medium (DMEM) with 10% fetal bovine serum (FBS). Cells were transfected with a *Renilla* luciferase reporter (with 5X-BoxB 3′ UTR or with the polyA protected 5xBoxB-A114-N40-Hammerhead (HhR) , alongside a firefly luciferase control and a tethering construct (115) (pCI-λN-HA-LacZ, -CNOT7, -PieF, or -BTG1) using the Lipofectamine 2000 manufacturer’s protocol (ThermoFisher) (using 800 ng endotoxin-free DNA at a 1:1:1 ratio and 2 µL Lipofectamine in Opti-MEM) and grown at 37°C and 5% CO_2_ for 16–20 h. Expression of the *Renilla* luciferase and firefly luciferase was analyzed using the Promega Dual-Luciferase Reporter Assay System (Promega). Cells were lysed in the kit’s passive lysis buffer for 1 h, and 2.5 µL of lysate was analyzed according to the manufacturer’s protocol (Promega). Luminescence was measured using an Infinite 200 PRO Mplex plate reader (TECAN) with a 10,000 ms integration time. *Renilla* luciferase activity is normalized to firefly luciferase for each sample, and the ratio is plotted relative to the LacZ control. The luciferase activity results represent three independent biological replicates.

### Transfection of HA-mCherry vectors into stable cell lines for AP-MS

Stable cell lines were generated using the Flp-In™ T-REx™ 293 system (ThermoFisher) by seeding a six-well plate with 4.8 × 10^5^ cells/well. The following day, cells were transfected with 2.5 µg pOG44 (Thermofisher), 200 ng pcDNA5 3xFLAG-CNOT7 or empty vector in 250 µL Opti-MEM (ThermoFisher) with 10 µL of Lipofectamine 2000. On day 2, cells were re-plated to 10 cm dishes. On the third day hygromycin B was added to a final concentration of 200 µg/mL to select for stable integrants. DMEM media was changed every three days until the cells were confluent. For CNOT7 induction before FLAG IP-MS, cells were seeded and grown to approximately 60-70% confluency in DMEM medium supplemented with 10% FBS (Wisent) in 15 cm dishes. Cells were then induced with 1 µg/mL of tetracycline (BioBasic) and transfected with 145 µL Lipofectamine 2000 (ThermoFisher) containing 58 µg DNA of pDEST53-HAmCherry-pieF or empty vector control as described above. After 24 h, cells were visually inspected for fluorescence by microscopy (Leica DMi8 inverted fluorescent microscope) to confirm the expression of each effector before harvest. Cells were harvested by washing once with 10 mL of PBS and pelleted at 100 *× g* for 5 min. The cell pellets were frozen in liquid nitrogen, weighed, and stored at −80°C until further processing.

### Immunoprecipitation of FLAG-tagged CNOT7 and preparation for mass spectrometry

Cell pellets from three independent 15 cm dishes were prepared as biological replicates. Pellets were lysed at 4°C in lysis buffer (50 mM HEPES-NaOH (pH 8.0), 100 mM NaCl, 2 mM EDTA, and 10% glycerol with freshly added 0.1% NP-40, 1 mM DTT, 1 mM PMSF, and protease inhibitor cocktail (1:500). Lysis buffer was added in a 1:4 pellet weight to volume ratio. Freeze fracturing was performed by freezing tubes in a dry ice ethanol bath and then thawing slowly in a 37°C water bath. After thawing, samples were placed back on ice and sonicated with three bursts at an amplitude of 25% for 5 s with 3 s of rest in between using a Q500 Sonicator with a 1/8” microtip (Qsonica,). Samples were then digested with 250U benzonase (MilliPoreSigma) for 30 min at 4°C with end-over-end rotation to reduce nucleic acid-mediated interactions. Lysates were cleared at 20,000 *× g* for 20 min at 4°C. Clarified lysates were normalized by volume and incubated with 60 µL of a 33% magnetic anti-FLAG M2 bead slurry (20 µL beads) (MilliPoreSigma) equilibrated in lysis buffer. Immunoprecipitation proceeded for 3 h at 4°C with end-over-end rotation. Following incubation, the magnetic beads were pelleted at 1,000 *× g* for 30 s at 4°C, magnetized, and lysate was removed. The beads were washed with 1 mL of lysis buffer at 4°C and transferred to new tubes, followed by a 1 mL lysis buffer wash and 1 mL ammonium bicarbonate buffer (50 mM, pH 8.0) wash. After washing, the beads were resuspended in 10 µL ammonium bicarbonate (50 mM, pH8.0) with 1 µg trypsin from porcupine pancreas (MilliPoreSigma) and incubated at 37°C overnight with rotation. The supernatant was collected, and a second round of digestion on beads was performed with 0.5 µg of trypsin in 5 µL of ammonium bicarbonate buffer (50 mM, pH 8.0) and rotated for 4 h at 37°C. Digestion was stopped by the addition of formic acid to a final concentration of 2.5%.

### Mass spectrometry acquisition using TripleTOF mass spectrometers

IP-MS samples were subjected to mass spectrometry in three biological replicates. A quarter of each sample was loaded at 800 nL/min onto a 15 cm 100 µm ID emitter tip packed in-house with 3.5 µm Reprosil C18 (Dr. Maisch GmbH, Germany). The peptides were eluted from the column at 400 nL/min over a 90 min gradient generated by a 425 NanoLC (Eksigent) and analyzed on a TripleTOF 6600 instrument (AB SCIEX). The gradient started at 2% acetonitrile with 0.1% formic acid and increased to 35% acetonitrile over 90 min followed by 15 min at 80% acetonitrile, and then 15 min at 2% acetonitrile for a total of 120 min. After each sample, the column was flushed twice for 1 h at 1,500 nL/min using an alternating sawtooth gradient from 35% acetonitrile to 80% acetonitrile. Each concentration gradient was maintained for 5 min. Column and instrument performance was verified after each sample by analyzing a 30 fmol bovine serum albumin (BSA) tryptic peptide digest with a 60 fmol α-casein tryptic digest in a short 30 min gradient. Mass calibration for the mass spectrometer was performed on the BSA reference ions between samples. Acquisition of ions was performed in data-dependent mode and consisted of one 250 ms MS1 TOF survey scan from 400 to 1250 Da followed by 20 100 ms MS2 candidate ion scans from 100 to 2,000 Da in high-sensitivity mode. Ions with a charge of 2+ to 4+ exceeding the threshold of 200 counts/s were selected for fragmentation. Former precursors were excluded for 10 s after one occurrence,

### MS peptide analysis and SAINT filtering

Data from the mass spectrometer were analyzed using the ProHits laboratory information management system (LIMS) platform (150). Wiff files were converted to MGF format using WIFF2MGF. MGF files were then converted to mzML format with ProteoWizard (3.0.4468) and AB SCIEX MS Data Converter (V1.3 beta), MzML files were searched using Mascot (v2.3.02) and Comet (2014.02 rev.2) (154). Search results were concatenated and analyzed with the Trans-Proteomic Pipeline (TPP) *via* the iProphet pipeline. The spectra were searched against a total of 72,518 proteins consisting of the NCBI human RefSeq database (version 57; 27 March 2020; forward and reverse sequences), *Legionella pneumophila* subsp. *pneumophila* (strain Philadelphia 1/ATCC 33152/DSM 7513) Lpg1972 (PieF) along with common contaminants from MaxQuant (155) and the Global Proteome Machine (http://www.thegpm.org/crap/index.html), as well as sequences from common fusion proteins and epitope tags. Database parameters were adjusted to search for tryptic cleavages, allowing up to two missed cleavage sites per peptide, MS1 mass tolerance of 40 ppm with charges of 2+ to 4+ and an MS2 mass tolerance of ±0.15 amu. Asparagine/glutamine deamidation and methionine oxidation were selected as variable modifications. A minimum iProphet probability of 0.95 was required for protein identification. Proteins detected with a minimal number of two unique peptides were used for protein interaction scoring. Significance Analysis of INTeractome (SAINTexpress version 3.6.1) was applied to calculate the probability of potential interactions from the background (156). Three biological replicates of a cell line expressing only the FLAG tag were subjected to the same process as FLAG–CNOT7 cell lines to function as a negative control. Each biological replicate was analyzed independently against the control before averaging biological replicate results and calculating Bayesian false discovery rates (BFDR). High-confidence interactions were those with BFDR ≤1%.

### *S. cerevisiae* kinetic growth curve assays

Cultures of wild-type BY4741 carrying pAG416GPD-pieF, lpg2149, or an empty vector control and BY4741 *pop2∆::KanMX* carrying an empty vector control were grown to saturation in 3 mL of SC-Ura with glucose at 30°C. The cultures were diluted to an OD_600_ of 0.05 in SC-Ura with or without 250 µg/mL of 6-azauracil (MilliporeSigma) in 200 µL in a 96-well plate. The plate was sealed a Breathe-Easy membrane (MilliporeSigma) and incubated at 30°C using an S&*P* growth curve robot (S&*P* Robotics Inc) with 15 min read intervals at OD_620_. Results were plotted using the R package ggplot2 (V3.3.5) and are representative of two biological replicates.

### Fluorescence microscopy

Imaging was performed using a Leica DMi8 inverted fluorescent microscope (Leica Microsystems). HEK293T cells were transfected with pT7-EGFP-C1-HsNot7 and pDEST53-HAmCherry-pieF, lpg2149, or empty vector control using Lipofectamine 2000 according to the manufacturer’s protocol (800 ng DNA, 2 µL Lipofectamine 2000 in OptiMem) and seeded at 1 × 10^5^ cells on poly-L-lysine coated glass coverslips in 24-well plates in DMEM with 10% FBS immediately following transfection. After 16–20 h at 37°C with 5% CO_2_, cells were washed gently three times with PBS. Cells were fixed in 4% paraformaldehyde (Electron Microscopy Sciences) for 20 min at RT and washed three times with PBS. The cells were permeabilized in PBS + 0.5% Triton X-100 for 5 min at RT and washed three times with PBS before mounting the coverslips onto microscope slides with Prolong GOLD Anti-fade with DAPI (Life Technologies). The mounted slides were allowed to cure for 24 h in the dark at RT. Nuclear exclusion statistics were calculated as a Student’s *t*-test between each sample.

## Data Availability

CCR4–NOT subunit composition data have been deposited as a complete submission to the MassIVE repository (https://massive.ucsd.edu/ProteoSAFe/static/massive.jsp) and assigned the accession number MSV000089325. The ProteomeXchange accession number is PXD033482.

## SUPPLEMENTAL MATERIAL

Tables S1 to S5 msphere.00891-24-s0001.xlsx BLI data, plasmid and primer information, ProHits filtering, and raw Y2H data.

ASM does not own the copyrights to Supplemental Material that may be linked to, or accessed through, an article. The authors have granted ASM a non-exclusive, world-wide license to publish the Supplemental Material files. Please contact the corresponding author directly for reuse.
